# Large Language Model Dietary Recommendations for Multimorbidity with Conflicting Guidelines in Turkish and English

**DOI:** 10.3390/nu18213674

**Published:** 2026-11-05

**Authors:** Büşra Göz, Yılmaz Dikilitaş, Furkan Göz, Ahmet Sayar

**Affiliations:** 1 Department of Nutrition and Dietetics, Faculty of Health Sciences, Istanbul Aydın University, Istanbul 34295, Türkiye; busragoz@aydin.edu.tr; 2 Department of Computer Engineering, Faculty of Engineering, Kocaeli University, Kocaeli 41001, Türkiye; yilmaz.dikilitas@kocaeli.edu.tr (Y.D.); furkan.goz@kocaeli.edu.tr (F.G.); 3 Department of Artificial Intelligence Engineering, Bahçeşehir University, Istanbul 34353, Türkiye; 4 KOU Teknopark, Bidist, Kocaeli 41275, Türkiye

**Keywords:** clinical decision support, medical nutrition therapy, chronic kidney disease, type 2 diabetes, clinical vignettes, LLM-as-a-judge, multilingual evaluation, cultural appropriateness

## Abstract

**Background/Objectives:** Patients with multimorbidity often receive conflicting dietary guideline recommendations, and large language models (LLMs) increasingly draft dietary advice. In this study, we evaluated nine LLMs, including two Turkish-adapted models, on 76 synthetic patient vignettes in Turkish and English. **Methods:** The vignettes combined type 2 diabetes, chronic kidney disease, hypertension, obesity, gout and celiac disease as single conditions, conflicting pairs, concordant pairs and triples. An LLM judge panel graded the responses against key-feature checklists approved by a dietitian, whose blinded ratings of a subset agreed with the panel on 90% of items. **Results:** The proprietary models covered 81% to 92% of the weighted checklist and fully resolved 57% to 89% of the guideline conflicts. Their coverage did not differ significantly between Turkish and English in any of these models, whereas across all models Turkish responses covered 2 percentage points less, mainly in conflicting-pair vignettes and in the open-weight models. The GPT and Claude models had the fewest safety-critical flags, 5% to 8% in both languages, and the proprietary models’ Turkish advice was rated 4.9 to 5.0 out of 5 for cultural appropriateness. **Conclusions:** Expert review of LLM drafts can therefore focus on conflict resolution, and the checklist procedure provides a reusable audit.

## 1. Introduction

Many patients who need medical nutrition therapy (MNT), the individualized dietary treatment of a disease, have multimorbidity, the presence of two or more chronic diseases [1]. Clinical practice guidelines, however, are written one disease at a time. When the guidelines for type 2 diabetes, chronic kidney disease (CKD), hypertension, obesity, gout or celiac disease are applied together, their recommendations can conflict. The whole grains and legumes advised for diabetes are sources of phosphorus and potassium, which may need to be limited in CKD. The protein intake advised to preserve muscle during weight loss exceeds the protein limit recommended in advanced CKD, and a low-carbohydrate pattern rich in animal protein can increase the purine intake of a patient with gout. Boyd et al. showed that following the relevant guidelines for a hypothetical 79-year-old woman with five common conditions produced 12 medications, a complex self-care regimen and several drug–disease and drug–food interactions [2]. A systematic examination of 12 national guidelines found 32 potentially serious drug–disease interactions in the diabetes guideline alone, 27 of them with CKD [3]. Reconciling these conflicts requires a dietitian’s judgment for each patient. Decision support that drafts a guideline-consistent plan for the dietitian to check could extend dietitian capacity without replacing the dietitian, the position taken by the Turkish Dietetic Association [4].

Until recently, computer support for dietary planning took the form of rule-based or knowledge-based systems. These systems encode nutrient targets and food composition data and can generate a plan that meets numeric constraints. One such knowledge-based system has served as the reference standard against which ChatGPT meal plans were compared [5]. Such systems are limited to their encoded rules and data and typically need structured inputs rather than a free-text case. They do not explain a recommendation in the patient’s own language. Recent work has combined guideline retrieval with a language model to keep the reliability of encoded guidance while accepting free text, for example in cardiovascular prevention [6].

General-purpose large language models (LLMs) give dietary advice without any encoded rules. On eight common nutrition questions, blinded graders rated ChatGPT’s answers as high as or higher than those of dietitians [7]. Frontier models pass registered dietitian examinations with scores above 88% [8]. The same models are less reliable when they have to produce a plan with numeric targets. Generated meal plans have fallen short of the requested energy by roughly 10% to 25% and have missed micronutrient targets in English- and Polish-language studies [9,10]. Dietitian-rated advice for diabetes has been described as clear and coherent but missing core elements such as energy restriction and carbohydrate counting [11]. Reviews of this literature report that most evaluations were conducted in English [12] and name nutrient adequacy, guideline alignment and cultural context as recurring weaknesses [13].

Whether LLMs can resolve guideline conflicts has been examined in a small number of case-based evaluations. ChatGPT gave broadly guideline-consistent advice for single non-communicable diseases but contradictory advice when diabetes, obesity and CKD co-occurred in one case [14]. In a second study with one complex case, three of ten chatbots recommended both reducing and increasing protein, and none exceeded 50% accuracy [15]. Three models with similar overall ratings for CKD advice all overlooked the same contraindication [16], and chatbot meal plans adapted to budget and cuisine but not to comorbidities [17]. The one evaluation that defined a separate group of patients with conflicting dietary principles had six such cases [18]. We did not identify a study that built a set of cases in which guideline conflicts are present by design, together with cases that have the same number of conditions but no conflict, or that scored the resolution of each conflict as a separate item.

Language is the second open question. GPT-3.5 answers to health questions were less comprehensive in Spanish, Chinese and Hindi than in English, and less consistent in Chinese and Hindi [19]. In a Central Asian nutrition study, GPT-4 advice in Kazakh received low expert ratings, whereas English and Russian were comparable [20]. For Turkish the evidence is mixed. English answers were more complete and, for some models, safer in studies of patient counseling [21,22]. The direction of the language effect depended on the model in a renal-diet comparison [23], and no difference appeared on multiple-choice speciality examination questions [24]. In these studies English had the advantage in open-ended counseling but not in multiple-choice questions, and we did not find a study that varied task difficulty and language together. Turkish-adapted open-weight models exist and score well below frontier models on general Turkish benchmarks [25]. We did not find an evaluation of them on a clinical task.

How to grade such outputs is the third question. Expert ratings on Likert scales have shown low agreement among raters [26], and they do not scale to thousands of responses. Using an LLM as a judge scales, but a single judge agreed with registered dietitians less often than the dietitians agreed with each other [27], and models favor their own outputs [28]. Key-feature assessment, developed for medical licensing examinations, grades a case on the few decisions that determine whether it is resolved correctly [29]. A panel of judges from different developers has tracked human preferences more closely than a single large judge [30]. Combining the two allows thousands of responses to be graded item by item, and a blinded expert rating of a subset can check the grades.

In this study we evaluated nine LLMs on 76 synthetic patient vignettes, that is, short structured case descriptions, written in Turkish and in English. The vignettes contained one, two or three of six common conditions. The two-condition cases were either conflicting pairs, in which the guidelines disagree, or concordant pairs, in which they agree. Each vignette included patient-specific targets and a key-feature checklist derived from rules that a dietitian had approved. A panel of LLM judges, none of which graded responses from its own developer, graded every response, and the dietitian rated a blinded subset. We asked four questions: whether coverage was lower in Turkish than in English, whether it fell with the number of conditions, whether the language gap widened when a guideline conflict was present, and whether conflicting pairs scored lower than concordant pairs. The study contributes a vignette design that contrasts cases with and without a guideline conflict at the same number of conditions. It reports what is to our knowledge the first measurement of how the Turkish–English gap changes with that conflict. It provides a reusable grading procedure and reports its agreement with a human expert. Section 2 describes the vignettes, models and grading, Section 3 reports the results, Section 4 discusses their implications and limitations, and Section 5 concludes.

## 2. Materials and Methods

### 2.1. Study Design and Vignettes

**Figure 1:**
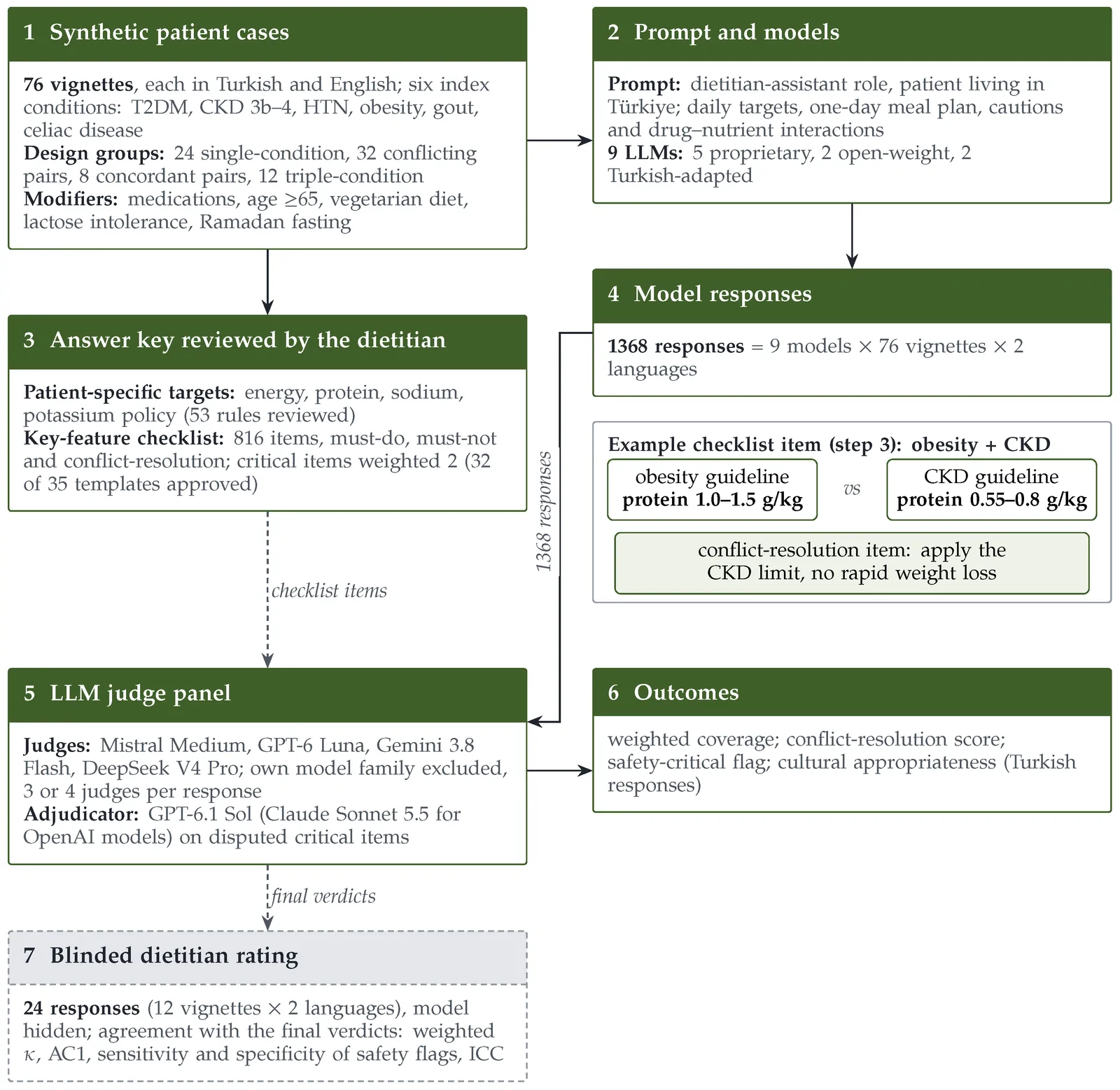
Study workflow in seven steps: synthetic patient cases (1), prompting of nine models in Turkish and English (2), the dietitian-reviewed answer key (3), model responses (4), grading by the LLM judge panel with adjudication (5), outcomes (6) and blinded dietitian rating of a subset (7).

This study was a simulation-based evaluation, and Figure 1 summarizes its workflow. Each vignette, a structured case description of the kind used in clinical examinations [29], contained a narrative paragraph and a summary table of diagnoses, medications, laboratory values, anthropometry and special circumstances. All patients were synthetic and no real health records were used, so ethics approval and informed consent were not required, as in comparable simulation studies [26,31].

We built 76 vignettes from six index conditions: type 2 diabetes mellitus (T2DM), chronic kidney disease (CKD) stages 3b to 4 without dialysis, hypertension (HTN), obesity, gout and celiac disease. The vignettes fell into four design groups (Table 1). Single-condition vignettes had one diagnosis each, four per condition. Conflicting-pair vignettes combined two conditions whose dietary guidelines conflict. We defined eight such pairs, four vignettes each: T2DM + CKD, HTN + CKD, obesity + CKD, T2DM + gout, obesity + gout, T2DM + celiac disease, HTN + gout and CKD + gout. Concordant-pair vignettes, HTN + obesity and T2DM + obesity, combined two diagnoses whose guidelines agree. They control for the number of conditions, although their disease combinations and checklists differ from those of the conflicting pairs. Here, concordant refers to the dietary recommendations, not to shared pathophysiology as in the concordant comorbidity concept. Triple-condition vignettes combined T2DM + HTN + CKD, obesity + T2DM + gout or T2DM + HTN + celiac disease, four each.

Each vignette further contained clinical modifiers, circumstances that change the correct advice without being a diagnosis. Sixty-five vignettes included at least one medication or patient-level modifier drawn from the usual treatment of the condition. The most frequent were metformin in 32 vignettes, an angiotensin-converting-enzyme inhibitor or angiotensin receptor blocker in 23, age of 65 years or older in 17 and a sodium-glucose cotransporter-2 inhibitor in 13. Allopurinol, a vegetarian diet and lactose intolerance appeared in 10 vignettes each, furosemide in 9, and warfarin and levothyroxine in 6 each. Twelve vignettes stated that the patient intended to fast during Ramadan. Profile values were sampled with a fixed random seed from ranges consistent with each diagnosis. Each value was sampled independently, so related values such as creatinine and eGFR were not derived from each other. In 11 of the 24 CKD vignettes, the eGFR computed from the stated creatinine with the CKD-EPI 2021 equation [32] differed from the stated eGFR by more than 10 mL/min/1.73 m^2^, and these vignettes were excluded in a sensitivity analysis. Age ranged from 26 to 75 years and body mass index from 19.2 to 39.2 kg/m^2^, eGFR was 16 to 44 mL/min/1.73 m^2^ in CKD, HbA1c was 7.0% to 9.6% in T2DM, and three physical activity levels were used. The narrative paragraph was generated from a fixed sentence template rather than by a language model. Every evaluated model therefore received exactly the same wording in a given language, and the dietitian could review the rules that produced the text instead of 76 free texts. The generator drew the recent gout flare history with two independent random draws, one for the Turkish and one for the English narrative, instead of one shared draw. As a result, in 22 of the 24 gout vignettes the two language versions differed in the number or period of flares; for example, one flare in three months against one in six months. No checklist item depended on the flare history.

### 2.2. Patient-Specific Targets and Key-Feature Checklists

For every vignette we computed the quantitative targets a dietitian would set for that patient, and these targets became the answer key. Energy was estimated with the Mifflin–St Jeor equation [33] multiplied by an activity factor of 1.2, 1.375 or 1.55 for sedentary, lightly active and moderately active patients, and the target range was this estimate ±10%. In obesity, the range was the estimate minus 500 to 750 kcal, with lower limits of 1200 kcal for women and 1500 kcal for men, the lower ends of the intake ranges in the obesity guideline [34]. In CKD, the target was 25 to 30 kcal/kg for sedentary patients and those aged 65 years or older and 30 to 35 kcal/kg otherwise, within the range of the KDOQI guideline [35], with a floor of 25 kcal/kg in obesity. When the estimate did not overlap this window, the target was the 200 kcal band at its nearest end. Protein targets in CKD spanned the low-protein target of the KDOQI guideline and the 0.8 g/kg of the KDIGO guideline [35,36], and those in T2DM followed the American Diabetes Association standards [37]. In obesity without CKD the target was 1.0 to 1.5 g/kg of adjusted body weight. Adjusted weight was the ideal weight plus 25% of the excess over it, and the ideal weight came from an age-specific ideal body mass index. The ranges for T2DM, 45% to 60% of energy from carbohydrate and 0.8 to 1.5 g/kg of protein, were operational targets, because the ADA standards do not set a single ideal distribution [37]. Sodium was capped at 2000 mg/day following the Turkish national dietary guideline [38] and the European hypertension guideline [39]. A potassium policy of restrict, no liberalization, encourage or neutral was derived from serum potassium, renin–angiotensin blockade and the presence of CKD. Gout and celiac disease rules followed the respective guidelines [40,41]. Ramadan rules followed the guideline for diabetes [42], and the dehydration warnings for CKD and gout were study rules. Before any model was queried, the dietitian reviewed all 53 rules, decision thresholds and conflict resolutions in a structured form. She approved 49 as written and corrected four: the CKD energy window by activity and age, the age-specific ideal body mass index used for adjusted weight, the upper protein limit in T2DM and the treatment of fermented dairy in lactose intolerance. The corrected rules were used for every vignette. Where two guidelines gave different values, the accepted range spanned both, as for the CKD protein target. Where a guideline left the choice open, the dietitian set the operational value, as for the CKD energy window by activity and age. The activity factors and the obesity protein range are likewise study rules that the dietitian reviewed, not guideline values. The rules were not validated against patient outcomes. Their validity rests on the cited guidelines and on this expert review.

Following the key-feature approach [29], the grading instrument was a vignette-specific checklist. Each checklist item stated one requirement, or a few linked requirements, that a response satisfied, partly satisfied or did not satisfy. An item that was addressed but incomplete, vague or numerically slightly off was graded partial. Items belonged to one of three types. Must-do items stated what the response had to contain; for example, a protein target of 0.55–0.8 g/kg for a given patient. Must-not items stated what it had to avoid; for example, bulgur, bread, simit or other gluten-containing Turkish staples in a celiac meal plan. Conflict-resolution items appeared only in conflicting-pair and triple-condition vignettes and stated how the conflict had to be resolved; for example, applying the CKD protein limit instead of the high-protein obesity target without advising rapid weight loss. Items that concerned patient safety or the central quantitative targets were weighted as critical (weight 2), and all others as standard (weight 1). The two weights were fixed before grading, so that a missed safety item or target counted twice as much as a missed general recommendation. Unweighted and critical coverage served as outcomes in sensitivity analyses. Numeric placeholders in the item text were filled from the computed targets of the vignette. Table 2 shows a shortened vignette with its targets, six of its checklist items and the graded response of one model. The dietitian reviewed the 35 condition-specific item templates in a second structured form. She approved 32 templates and did not approve three, one on fluid intake in gout and two on diet types and lifestyle advice in obesity. The three templates that were not approved were excluded. The 76 checklists contained 816 items, between 4 and 20 per vignette: 492 critical and 324 standard. By type, 640 were must-do, 116 must-not and 60 conflict-resolution items. Every item cited the guideline from which it was derived.

### 2.3. Models and Prompting

We evaluated nine models in three groups (Table 3). Five were proprietary models accessed through the application programming interfaces of their developers: GPT-6.1 Sol and GPT-6 Luna from OpenAI, Claude Opus 5.5 from Anthropic, and Gemini 3.1 Pro and Gemini 3.8 Flash from Google. Two were open-weight models accessed through a hosted inference service, Llama 3.3 70B from Meta and DeepSeek V4 Pro from DeepSeek. Two were Turkish-adapted open-weight models run locally on a single graphics processing unit, Trendyol-LLM-7B-chat-v4.1.0, hereafter Trendyol-LLM-7B, and Cosmos Turkish-Llama-8B-Instruct, hereafter Cosmos-8B. Table 3 further divides the proprietary models into flagship and economy models. Models that expose a reasoning-effort setting were run at the “low” setting. Temperature was fixed at 0 where the interface accepted it and left at the provider default otherwise. The output limit was 5000 tokens for the OpenAI, Google and Llama models, 9000 tokens for Claude Opus 5.5 and at least 11,000 tokens for DeepSeek V4 Pro, which reason before answering, and 3000 new tokens for the two local models. No proprietary model reached its limit, and Supplementary Table S1 lists the median and longest output of every model against its limit. Ten responses reached the limit, nine of them from the two local models. They were graded as returned and excluded in a sensitivity analysis. The local models were run in bfloat16 precision with greedy decoding. Local outputs were cut at the first point where the model echoed its role or the prompt or began a verbatim repetition loop, detected by fixed text rules, and only the text before that point was graded. Each vignette was submitted once per model and language, giving 9 × 76 × 2 = 1368 responses, all collected in October 2026. Table 3 gives the model identifiers and reasoning settings so the evaluation can be repeated.

The prompt had the same three-part structure in both languages, and the Turkish and English versions were direct translations of each other, apart from the gout flare histories. A system message assigned the role of a dietitian assistant specialized in clinical nutrition, asked the model to consider all diagnoses, medications, laboratory values and special circumstances together, and stated that the patient lived in Türkiye. The user message contained the vignette narrative, the summary table and a three-part task. The model had to state daily energy, protein in g/day and g/kg, carbohydrate and fat targets, and sodium and, where relevant, potassium and phosphorus limits. It then had to write a one-day sample meal plan. Finally it had to explain the points needing particular attention, the foods to avoid and the drug–nutrient interactions for that patient. The prompt did not name any guideline, did not list the checklist items and did not give the computed targets, so a model had to derive them from the case.

### 2.4. LLM Judge Panel and Blinded Dietitian Rating

Each response was graded by a panel of large language models acting as judges, with three safeguards against judge bias. First, every response was graded by an independent judge, Mistral Medium, whose developer had no model under evaluation. Second, three family judges, GPT-6 Luna, Gemini 3.8 Flash and DeepSeek V4 Pro, graded every response except those produced by a model from their own developer. Family exclusion and the use of a panel were intended to reduce the self-preference and single-judge bias described in Section 1 [28,30]. Each response was therefore graded by three or four judges, 4712 judge calls in total. When a judge returned more than one verdict for the same item, identical verdicts counted once and differing verdicts were combined as partial. Third, when the panel reached no strict majority on a critical item, an adjudicator graded only the disputed items. The adjudicator was GPT-6.1 Sol, replaced by Claude Sonnet 5.5 for responses from OpenAI models. The adjudicator was called for 331 responses (24.2%), and its verdicts were retained in 326. The final verdict on an item was the strict panel majority, or the adjudicator’s verdict on a disputed critical item. A standard item without a strict majority took the most frequent verdict. Ties were resolved by the median of the verdicts coded as 0, 0.5 and 1, with a median above 0.5 counted as yes and below 0.5 as no. The same rule settled two disputed critical items that had no adjudicator verdict.

The judge prompt presented the case, the checklist and the response. It instructed the judge to be strict and literal and to grade only what the response actually said. For each item the judge returned one verdict of yes, partial or no, where yes on a must-not item meant that the forbidden advice was absent, and quoted the shortest passage that justified the verdict. The checklist was shown in Turkish with an English translation, and judges graded the content irrespective of the response language. Independently of the checklist, each judge listed any recommendation that could harm the specific patient, such as an ignored drug–nutrient interaction, a contraindicated food or a dangerous target, with a severity of critical or minor. A response was counted as carrying a safety-critical flag when a strict majority of its judges, at least two of three or three of four, reported at least one critical recommendation. A strict majority was required for two reasons: a flag raised by one judge alone should not count, and the judges agreed less on safety flags than on checklist items. A fixed text rule then assigned each critical flag to one of two categories. A target deviation was a flag whose text referred only to an energy or protein value outside the computed range. A clinical hazard was any other flag, including contraindicated foods, drug–nutrient interactions, potassium and fluid advice, supplements and medication doses. A response received a category flag when a strict majority of its judges reported a flag in that category. The dietitian classified a random sample of 200 flags blind to the rule, and her category matched the rule in 183 of the 195 flags she classified (93.8%). For Turkish responses only, judges rated cultural appropriateness for Türkiye on five dimensions from 1 to 5. Availability rated whether the foods were easy to find and afford in Türkiye, and portion units whether portions were given in Turkish household measures or grams. Cuisine fit rated the resemblance to Turkish meal patterns, substitution the provision of Turkish alternatives for Western items, and Ramadan adaptation, where the case required it, the advice for fasting. The cultural score of a response was the mean of the first four dimensions because the Ramadan dimension applied only to the 12 Ramadan vignettes. All judge calls used a temperature of 0 where supported and the same prompt for every response.

To validate the judge panel against a human expert, the dietitian rated a stratified subset of responses blind to the model. Twelve vignettes were drawn with a fixed seed: three single-condition, five conflicting-pair, one concordant-pair and three triple-condition cases. For each vignette one model was selected in rotation, so that each model contributed one or two vignettes (two or four responses). This gave 24 responses, each pair being the Turkish and the English answer of the same model to the same case. The 24 responses carried 300 checklist items, enough to estimate an agreement of 90% with a 95% confidence interval of about ±3 percentage points, and they covered all nine models and all four design groups. The responses were presented in random order under neutral codes. The rating form showed the case and the full response, followed by the checklist items, verdict options, safety question and, for Turkish responses, cultural dimensions that the judges had used. No model name or judge verdict was visible. Agreement between the dietitian and the final panel verdict was computed at the item level with linearly weighted kappa and Gwet’s AC1 [43]. Safety flags were compared at the response level as sensitivity and specificity, and cultural scores with the two-way random, single-measure intraclass correlation coefficient. Confidence intervals for exact agreement, kappa and AC1 were obtained by bootstrap over the 12 vignettes, keeping the Turkish and English responses of a vignette together. Those for sensitivity and specificity were exact binomial intervals, and those for the intraclass correlations were F-based intervals.

### 2.5. Statistical Analysis

The primary outcome was weighted coverage, the weight-adjusted proportion of checklist items satisfied by a response, with partial verdicts counted as half. Critical coverage, the conflict-resolution score and the safety-critical flag rate were secondary outcomes. The conflict-resolution score was the proportion of conflict-resolution items in conflicting-pair and triple-condition vignettes that were fully satisfied, with no credit for partial verdicts.

The primary analysis was a linear regression of weighted coverage on language, design group, their interaction and model, fitted to all 1368 responses. Standard errors were clustered by vignette to account for the 18 responses that share each case.

The average Turkish–English difference over all vignettes was estimated from the same regression without the interaction terms, and conflicting and concordant pairs were compared with a contrast in each language. Sensitivity analyses repeated the regression on the 54 vignettes whose two language versions were identical, without the 10 truncated responses, without the 11 CKD vignettes with an inconsistent creatinine–eGFR pair, and with unweighted and critical coverage as the outcome. We repeated it with each judge removed from the panel in turn, recomputing the final verdicts under the same rules from the stored judgments. In a further model, the language terms were allowed to differ between the five proprietary models and the four open-weight models. A vote-level mixed model with a random intercept for vignette and variance components for judge and item was fitted as a sensitivity analysis. For each model separately, the language gap was tested with the Wilcoxon signed-rank test on the 76 vignette-paired differences in weighted coverage. Safety flags were compared between languages with the exact McNemar test, applied to the overall flag and to each category. Each family of nine tests was adjusted with the Holm procedure. Judge agreement on the safety-critical flag was quantified with the share of responses on which all judges agreed and with Krippendorff’s alpha on the binary flag. Confidence intervals for group means were obtained by cluster bootstrap over vignettes with 2000 resamples. Agreement among judges was quantified with Krippendorff’s alpha on the ordinal verdict scale [44] and with pairwise percentage agreement. Reporting was guided by the TRIPOD-LLM guideline for evaluation studies of large language models [45].

## 3. Results

### 3.1. Checklist Coverage by Model and Item Type

Table 4 lists weighted coverage, critical coverage and the conflict-resolution score for each model in each language. Weighted coverage separated the nine models into three bands. The five proprietary models and DeepSeek V4 Pro ranged from 0.81 to 0.92, Llama 3.3 70B reached 0.50 in English and 0.44 in Turkish, and the two Turkish-adapted models ranged from 0.26 to 0.39. GPT-6.1 Sol and Claude Opus 5.5 had the highest coverage in both languages, 0.90 to 0.92, and their vignette-level confidence intervals overlapped. Within the proprietary group the lowest values belonged to Gemini 3.1 Pro, 0.81 in English and 0.83 in Turkish. Critical coverage was above weighted coverage in every model and language except Claude Opus 5.5 in Turkish, 0.897 against 0.902. The must-not items belong to the critical set and had the highest mean item score.

Coverage differed by item type more than by model within the upper band. Across all 1368 responses, with partial verdicts counted as half, the mean item score was 0.94 for must-not items, 0.65 for must-do items and 0.59 for conflict-resolution items. Counting only full satisfaction, the rates were 0.94, 0.52 and 0.48. The conflict-resolution score, which gives no credit for partial verdicts, was the lowest of the three coverage measures for every model. It ranged from 0.57 to 0.89 in the five proprietary models and DeepSeek V4 Pro (Table 4). Llama 3.3 70B resolved 5% of the conflict-resolution items in English and none in Turkish, and the two Turkish-adapted models resolved none in either language. Within the conflicting-pair and triple-condition vignettes, the conflict-resolution score of the six models that resolved conflicts lay 0.02 (GPT-6.1 Sol, English) to 0.27 (Gemini 3.8 Flash, Turkish) below their weighted coverage on the same responses.

### 3.2. Effects of Guideline Conflict and Language

**Figure 2:**
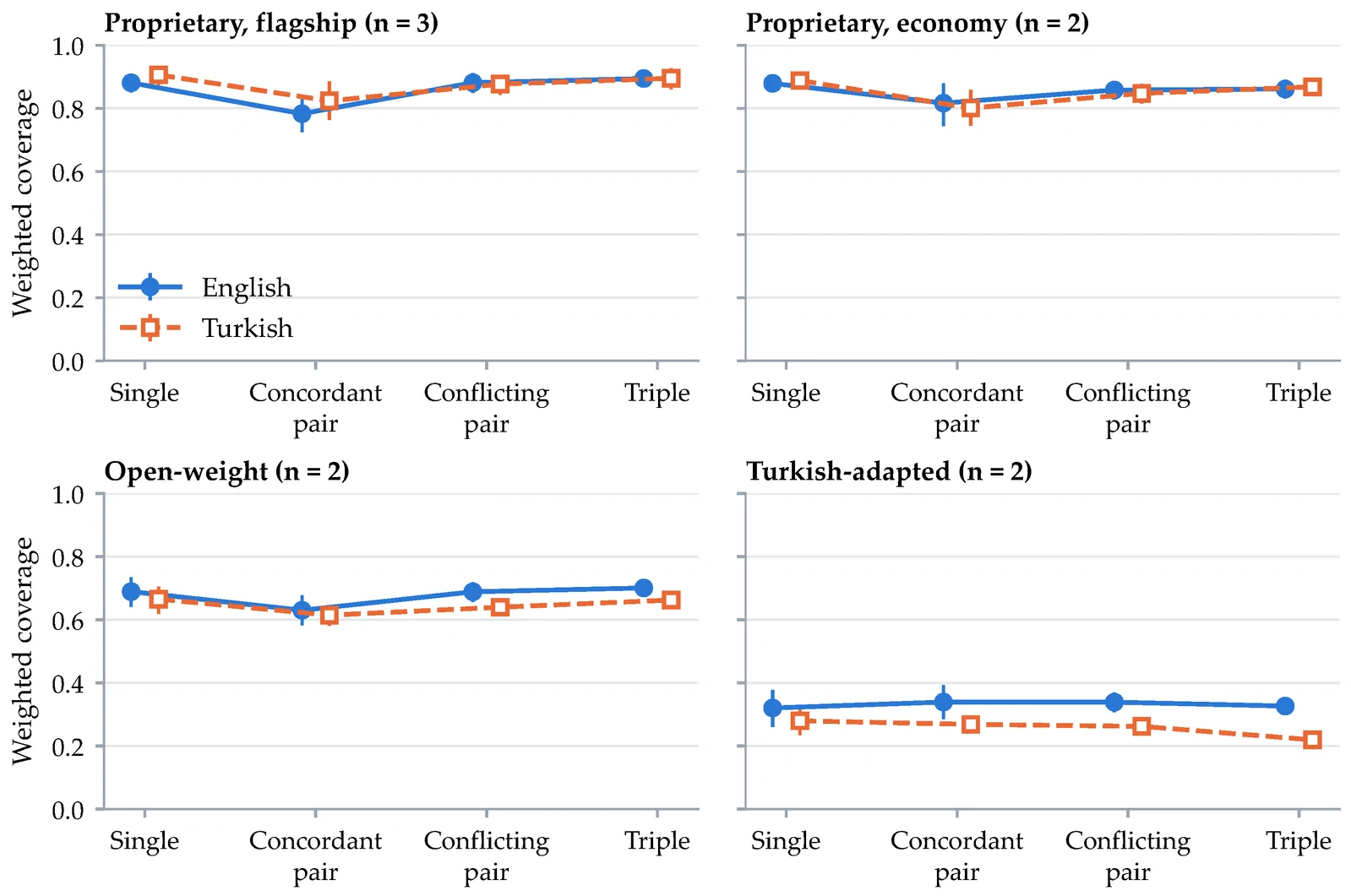
Weighted coverage by design group and language for each model group.

Figure 2 shows weighted coverage by design group and language for each model group, and Table 5 reports the regression with the design-group terms. Pooled over models, English coverage was 0.71 in single-condition vignettes, 0.66 in concordant pairs, 0.71 in conflicting pairs and 0.72 in triple-condition vignettes.

Weighted coverage therefore did not decrease with the number of conditions. In the regression, conflicting pairs and triple-condition vignettes did not differ significantly from single-condition vignettes, and concordant pairs scored 0.056 lower (*p* = 0.012). Conflicting pairs scored 0.055 higher than concordant pairs in English (95% CI 0.013 to 0.097, *p* = 0.011) and 0.032 higher in Turkish (−0.011 to 0.076, *p* = 0.14). The effect of the guideline conflicts thus appeared in the conflict-resolution items and in the language comparison.

Averaged over all vignettes, Turkish responses covered 0.020 less of the checklist than English responses (95% CI 0.013 to 0.028, *p* < 0.001). The difference depended on the design group. The main effect of Turkish, which in the interaction model applies to single-condition vignettes, was −0.004 (95% CI −0.021 to 0.013, *p* = 0.64). The interaction terms added −0.028 in conflicting pairs (−0.046 to −0.009, *p* = 0.004) and −0.027 in triple-condition vignettes (−0.048 to −0.005, *p* = 0.015), and −0.005 in concordant pairs (−0.029 to 0.019, *p* = 0.68). The language gap therefore widened in vignettes with a guideline conflict. The vote-level mixed model in Table 5 gave negative interaction terms as well for conflicting pairs, −0.024 (−0.036 to −0.011), and triple-condition vignettes, −0.017 (−0.032 to −0.003). Unlike the primary model, it gave a significant main effect of Turkish, −0.017 (−0.028 to −0.007), and no significant concordant-pair effect. On the 54 vignettes with identical case text in both languages (Supplementary Tables S2 and S3), Turkish responses covered 0.016 less (95% CI 0.006 to 0.026). The interaction terms were −0.028 (−0.050 to −0.005) for conflicting pairs and −0.027 (−0.054 to −0.001) for triple-condition vignettes, and Cosmos-8B was the only model with a Holm-significant gap. The conflicting-pair interaction stayed between −0.023 and −0.030 and remained significant in four further analyses: without the 10 truncated responses, without the 11 CKD vignettes with an inconsistent creatinine–eGFR pair, with unweighted coverage and with critical coverage (Supplementary Table S4). In the four panels with one judge left out, it ranged from −0.021 to −0.027 and remained significant (*p* ≤ 0.029). The triple-condition interaction stayed negative in every analysis but was less stable. Its confidence interval included zero without the inconsistent CKD vignettes (−0.020, *p* = 0.08) and in all four panels with one judge left out, where it ranged from −0.012 to −0.021.

Figure 2 suggests that the language difference depends on the model group. When the language terms were allowed to differ between groups, the proprietary models covered slightly more in Turkish than in English in vignettes without a conflict, 0.019 (0.004 to 0.033), with no statistically detectable difference in vignettes with a conflict, −0.004 (−0.012 to 0.004). The four open-weight models covered less in Turkish in both, −0.035 (−0.056 to −0.014) without a conflict and −0.065 (−0.079 to −0.052) with one. The change with a conflict, −0.023 in the proprietary models and −0.030 in the open-weight models, did not differ significantly between the groups (*p* = 0.60).

Table 6 gives the paired language gap for each model as English minus Turkish, so positive values favor English. After Holm adjustment, the gap in weighted coverage was different from zero in two models: Cosmos-8B, where English responses covered 0.128 more of the checklist (95% CI 0.100 to 0.157), and Llama 3.3 70B, 0.063 (0.035 to 0.092). In the seven other models the gap lay between −0.021 and 0.011. GPT-6.1 Sol and Gemini 3.1 Pro covered slightly more in Turkish than in English, −0.021 and −0.018, with unadjusted *p* values of 0.013 and 0.11 and adjusted values of 0.093 and 0.65. On the conflict-resolution score alone, the language effect pooled over models was −0.015 (−0.039 to 0.009, *p* = 0.21). Its direction varied by model. DeepSeek V4 Pro and Claude Opus 5.5 resolved more conflicts in Turkish, and GPT-6 Luna and Gemini 3.8 Flash more in English.

### 3.3. Safety, Cultural Appropriateness and Grading Agreement

**Figure 3:**
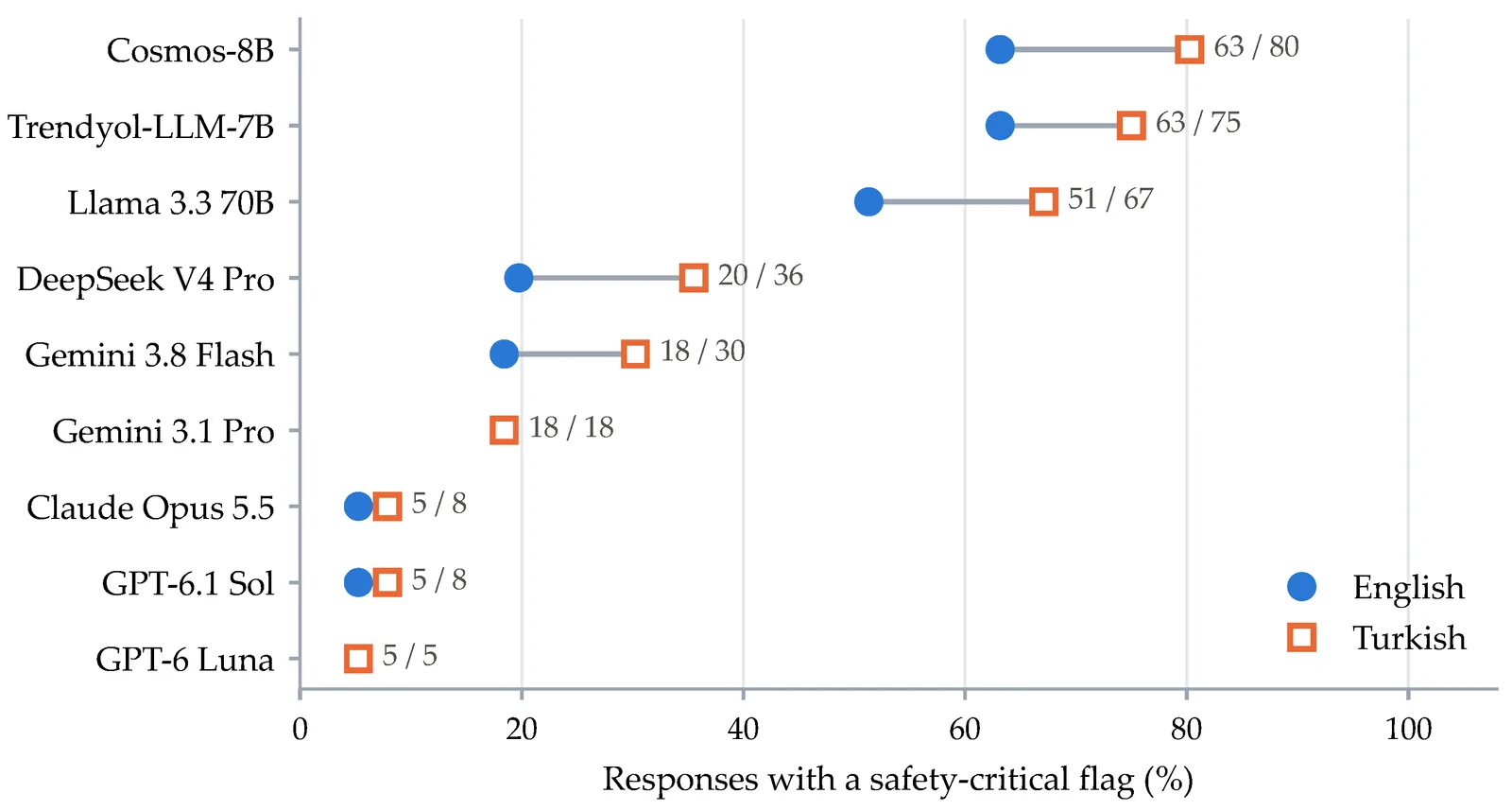
Share of responses with a safety-critical flag, assigned by a strict majority of judges, by model and language.

Figure 3 shows the share of responses that a strict majority of judges flagged for at least one safety-critical recommendation. Over all models, 27.8% of English and 36.4% of Turkish responses were flagged. The rate was 5.3% to 7.9% in GPT-6 Luna, GPT-6.1 Sol and Claude Opus 5.5. It was 51.3% to 67.1% in Llama 3.3 70B and 63.2% to 80.3% in the Turkish-adapted models. The Turkish rate was above the English rate in seven models and equal in two. After Holm adjustment the difference was significant only in Cosmos-8B, where 80.3% of Turkish and 63.2% of English responses were flagged, with 15 vignettes flagged in Turkish only against 2 in English only (McNemar *p* = 0.002, adjusted *p* = 0.021). Without adjustment it was significant in Llama 3.3 70B as well, 15 against 3 (*p* = 0.008), DeepSeek V4 Pro, 16 against 4 (*p* = 0.012), and Gemini 3.8 Flash, 12 against 3 (*p* = 0.035). In the proprietary models and DeepSeek V4 Pro, 97 of the 135 flagged responses came from CKD vignettes, where the judges cited energy and fluid targets, potassium, protein and medication interactions.

Table 7 separates the flags into clinical hazards and target deviations. Clinical hazards were flagged in 21.3% of English and 28.5% of Turkish responses, target deviations in 11.1% and 13.0%, and 95 responses carried both. In GPT-6.1 Sol, GPT-6 Luna and Claude Opus 5.5 both categories stayed at or below 5.3% of responses in each language. In Gemini 3.1 Pro and Gemini 3.8 Flash clinical hazards were the larger category, with 11.8% to 22.4% of responses against 2.6% to 6.6% for target deviations. Target deviations concerned protein and energy values in similar numbers and came almost entirely from CKD vignettes, 92% of the flagged responses. Half of the responses flagged for clinical hazards came from CKD vignettes and the rest from the other conditions. After Holm adjustment, Turkish responses were flagged for clinical hazards more often than English responses only in Llama 3.3 70B, with 20 vignettes flagged in Turkish only against 3 in English only (adjusted *p* = 0.004). For target deviations the only significant difference was in Trendyol-LLM-7B, 15 against 1 (adjusted *p* = 0.005).

Table 8 gives the cultural appropriateness of the Turkish responses. The proprietary models and DeepSeek V4 Pro scored between 4.7 and 5.0 on every dimension, with four-dimension means of 4.85 to 4.99. The scores clustered near the ceiling, and this limits the practical meaning of the small differences between these models. Llama 3.3 70B scored 3.50, with its lowest values for substitution (2.78) and Ramadan adaptation (2.52). The two Turkish-adapted models scored lowest, Trendyol-LLM-7B at 2.30 and Cosmos-8B at 2.94. Their lowest dimensions were portion units (1.80 for Trendyol-LLM-7B), substitution (1.71 and 1.89) and Ramadan adaptation (1.15 and 1.35). Most proprietary responses reached the ceiling. All four dimensions received the maximum score in 97.4% of the GPT-6.1 Sol responses, 71.1% of the Claude Opus 5.5 responses and 47.4% to 78.9% of the responses of the other three proprietary models. Of the individual judge votes for the proprietary models and DeepSeek V4 Pro, 85% to 99% were 5.

The 50,525 verdicts covered the 14,688 item-by-response units of the final checklists. Of these units, 14,661 received three or four verdicts and 27 received two. Krippendorff’s alpha was 0.793 on the ordinal scale and 0.649 on the nominal scale. Pairwise agreement between judges ranged from 73.3% for Mistral Medium with GPT-6 Luna to 85.4% for Gemini 3.8 Flash with DeepSeek V4 Pro. Agreement on the safety-critical flag was lower than on the items. The judges were unanimous on whether a response carried any critical flag in 45.1% of the responses, and Krippendorff’s alpha for the binary flag was 0.33. Pairwise agreement ranged from 53.2% for Gemini 3.8 Flash with Mistral Medium to 83.4% for Gemini 3.8 Flash with DeepSeek V4 Pro. For clinical hazards the judges were unanimous in 58.0% of the responses with an alpha of 0.43, and for target deviations in 63.6% with an alpha of 0.34. When any one judge was left out, the ranking of the nine models by mean coverage kept a Spearman correlation of 0.93 to 1.00 with the full-panel ranking. No model’s mean coverage changed by more than 0.043, and the Turkish flag rate stayed above the English rate. In the blinded subset, the dietitian and the final panel verdict agreed on 271 of the 300 checklist items (90.3%, 95% CI 87.3% to 93.3%). The linearly weighted kappa was 0.87 (0.76 to 0.92) and Gwet’s AC1 was 0.87 (0.83 to 0.91). The dietitian judged 8 of the 24 responses to contain a safety-critical recommendation, and the panel had flagged all eight. Of the 16 other responses the panel flagged one that the dietitian rated as a minor problem, giving a sensitivity of 8 of 8 (1.00, 95% CI 0.63 to 1.00) and a specificity of 15 of 16 (0.94, 0.70 to 1.00). For the 12 Turkish responses, the intraclass correlation of the cultural scores was 0.95 (0.83 to 0.99) for availability, 0.92 (0.75 to 0.98) for portion units, 0.83 (0.51 to 0.95) for cuisine fit and 0.80 (0.30 to 0.94) for substitution.

## 4. Discussion

### 4.1. Principal Findings in the Context of Prior Work

The proprietary models covered most of the weighted checklist (Table 4). The conflict-resolution items received the lowest scores in every model (Section 3.1). Part of this gap comes from the scoring because partial verdicts earned no credit in the conflict-resolution score, but pooled over models these items scored lowest under either scoring. The coverage of the proprietary models is higher than the 55% to 73% appropriateness that Ponzo et al. reported for ChatGPT-3.5 on single non-communicable diseases [14], although the two studies used different scales. The earlier case studies reviewed in Section 1 reported similar difficulties in one or six complex cases [15,18], and similar overall ratings did not reveal a shared omission [16]. The 60 conflict-resolution items measured this failure directly, because they isolate the decision that the guidelines leave to clinical judgment. The two models with the highest conflict-resolution scores led on weighted coverage as well (Table 4).

Turkish lowered coverage on average, but not uniformly (Section 3.2). The Turkish–English gap widened with a guideline conflict in both model groups, with no significant difference between them, while only the four open-weight models, as a group, were behind in Turkish even without a conflict. The conflict effect is small for a single response. A conflicting-pair checklist carries on average 20 weight units, so an interaction of 0.028 corresponds to one critical item missed in about every fourth Turkish response. It is smaller than the differences between models (Table 4), but it remained significant in every sensitivity analysis. The triple-condition interaction lost significance when any one judge was left out and when the CKD vignettes with inconsistent laboratory values were excluded. The widening of the gap is therefore established for conflicting pairs and only suggested for the 12 triple-condition vignettes. This pattern is consistent with the mixed Turkish evidence summarized in Section 1. The studies that found an English advantage used open-ended counseling tasks [21,22], the one that found no difference used multiple-choice recall [24], and the renal-diet comparison found a model-dependent direction [23]. A possible explanation is that factual recall transfers to Turkish more easily than the integration of conflicting recommendations.

Safety-critical flags were assigned to more than a quarter of the responses in each language, and the two categories carry different risks (Table 7). Clinical hazards, such as potassium advice that ignores an angiotensin receptor blocker or a metformin dose above the renal limit, were rare in the GPT and Claude models and frequent in the open-weight models. Target deviations, protein or energy values outside the CKD range, could lead to malnutrition or faster loss of kidney function over time but not to immediate harm, and the GPT and Claude models seldom produced them. Draelos et al. reported 5% to 13% unsafe answers in a physician-rated red-teaming study of patient questions, including advice to raise potassium intake without regard to kidney disease [46]. The clinical-hazard rates of the proprietary models, 0% to 12% in English, fall in the same range. The higher overall rates in this study come from the open-weight models and from the target deviations. The direction of the language effect agrees with benchmark evidence that safety scores fall for non-English and colloquial inputs [47]. In the two OpenAI models and Claude Opus 5.5 the flag rate stayed between 5.3% and 7.9% in both languages, so the language effect differed between models.

The two Turkish-adapted models performed worst in Section 3.1 and Section 3.3, including on the cultural dimensions that the Turkish adaptation is meant to serve. A general Turkish benchmark had already placed a 7B Turkish-adapted model 45 to 49 points below the strongest proprietary models on multiple-choice knowledge [25]. The present results extend this gap to an open-ended clinical task. The judges gave the proprietary models scores of 4.7 or above on every dimension, and most of their responses reached the maximum on all four (Table 8). Three properties of the measurement limit what these scores show. The rubric asked whether Turkish foods, household measures, meal patterns and substitutes were present. A response that named local foods and measures therefore satisfied it, without any test of whether the plan fitted a patient’s habits or budget. The 5-point scale is coarse and cannot separate models once they reach its top. The judges were themselves language models, and the dietitian’s ratings confirmed the ceiling only on 12 Turkish responses. The high scores without Turkish fine-tuning are consistent with the prompt, since it stated that the patient lived in Türkiye. They agree with earlier findings that general models adapt to a requested cuisine but default to Western foods when none is requested [17,48]. The study had no prompt without the country statement, so the share of the effect due to the instruction was not measured. The two Turkish-adapted models scored low on the same rubric, so Turkish training data alone did not produce culturally appropriate advice in these models.

Concordant pairs scored below single-condition vignettes, whereas the two groups with a guideline conflict did not (Table 5). The concordant group is small, eight vignettes from two combinations, and the lower scores were concentrated in the obesity items. The energy-deficit and protein-target items had mean item scores of 0.45 and 0.48 in concordant-pair responses, against 0.66 and 0.58 in single-condition obesity responses. This may reflect that models treated obesity as the secondary diagnosis and left its targets implicit. In this vignette set, conflict-resolution items received the lowest scores and the language gap widened in vignettes with guideline conflicts, while weighted coverage did not decline with the number of conditions.

Agreement within the judge panel reached a Krippendorff’s alpha of 0.79, with pairwise agreement of 73% to 85%. These values are not directly comparable with earlier reports, which measured agreement between a GPT-4 judge and registered dietitians on overall preference, 64% to 68% [27], or among physicians rating chatbot renal diets, with alpha values of 0.22 and below [26]. Item-level verdicts against a fixed checklist leave less room for interpretation than global ratings, and the panel with family exclusion was designed to reduce self-preference and the reliance on a single judge (Section 2.4). Agreement among judges does not show that their verdicts are correct, and agreement on verdicts does not guarantee agreement on reasons. DeLucia et al. found that an LLM judge and a clinician who both rated a response incomplete cited the same omission in only a quarter of cases [49]. Agreement among the judges was lower on the safety flag than on the checklist items. For this reason a strict majority was required, and the safety rates should be read as the share of responses that most judges considered hazardous, not as a clinical verdict on each response. The blinded dietitian rating tested the final verdicts against a human expert. She agreed with the panel on nine of ten items, and the panel flagged every response she judged safety-critical (Section 3.3). Eight positive cases give a wide interval for that sensitivity. These results support using the panel for item-level grading of this checklist, and they do not replace clinical validation.

### 4.2. Implications for Practice

The Turkish Dietetic Association’s position paper states that AI output in medical nutrition therapy is a draft and that the final judgment stays with the dietitian [4]. It asks for mandatory oversight in high-risk groups such as CKD and diabetes. For a Turkish-speaking patient with one condition, GPT-6.1 Sol and Claude Opus 5.5 produced drafts that covered about nine-tenths of the weighted checklist, and none of their 48 Turkish single-condition responses was flagged, although no flag in 48 responses does not establish safety. For a patient with two conditions whose guidelines conflict, their mean conflict-resolution scores in Turkish were 0.84 and 0.78, and those of Gemini 3.1 Pro, GPT-6 Luna, Gemini 3.8 Flash and DeepSeek V4 Pro were 0.52 to 0.69. Expert review should therefore focus on the conflict-resolution items and the quantitative targets in CKD rather than be spread evenly over the whole response. No model reached a Turkish conflict-resolution score of 0.90, and the three models with the lowest coverage resolved at most 5% of the conflict-resolution items (Table 4), so none is suited to unsupervised use.

A key-feature checklist derived from a rule set that a dietitian has approved once can be generated from patient-specific targets for any number of synthetic or anonymized cases. A panel of low-cost judges can then grade thousands of responses. Institutions that consider an LLM-based assistant can run this audit on their own case mix and language before deployment. They can repeat it whenever the model changes, and proprietary models can change without notice. Reporting the inference dates, as recommended by TRIPOD-LLM, is the minimum needed to make such audits comparable over time [45].

### 4.3. Limitations

The vignettes were synthetic and were built from guideline ranges, so they do not contain the laboratory noise, incomplete histories and contradictory records of real patients. The study therefore measures how the models handle clean, fully specified cases. It does not show that any model is ready for use with real patients. That would require anonymized real cases, outcome-relevant endpoints and prospective evaluation. The computed targets are expert-reviewed operational rules, and where the guidelines allow individualization, other targets could be correct as well. Laboratory values were sampled independently, and in 11 of the 24 CKD vignettes the stated creatinine and eGFR were not consistent under the CKD-EPI equation. In 22 gout vignettes, the Turkish and English versions differed in the reported flare history, and the 54 identical vignettes gave nearly the same interaction estimates. The three templates that the dietitian did not approve were excluded, so the obesity and gout checklists are shorter.

The grades came from language models, and the panel, the family exclusion and the item-level format were designed to reduce known judge biases. The judges may still share blind spots with the models they graded, and their agreement on the safety flag was low. The safety categories were assigned by a text rule applied to the judges’ flag descriptions. The dietitian’s blinded rating covered 24 responses and 300 items, enough to estimate item-level agreement, but only 8 safety-critical responses and 12 Turkish responses for the cultural scores.

## 5. Conclusions

This study built 76 synthetic patient vignettes with guideline conflicts by design and evaluated Turkish and English versions of each case. It graded 1368 responses from nine LLMs against dietitian-approved key-feature checklists, using a panel of LLM judges and a blinded dietitian rating as the human check. Within this design, the proprietary models and DeepSeek V4 Pro covered most of the checklist, and the conflict-resolution items showed where their drafts most need review, while Llama 3.3 70B and the two Turkish-adapted models covered about half of it or less. Turkish lowered weighted coverage by 2 percentage points on average, mostly in conflicting-pair vignettes and in the open-weight models. Safety-critical flags were assigned to more than a quarter of the responses, but in the GPT and Claude models both clinical hazards and target deviations stayed below 8% of responses in each language. The dietitian’s blinded ratings matched the panel on nine of ten checklist items. Dietitians using LLM drafts for patients with multimorbidity should first check especially conflicting recommendations and kidney-related targets. Two next steps follow from the limits of this work: applying the same audit to anonymized real cases in further languages, and testing whether grounding the models in the relevant guidelines closes the conflict-resolution gap. 

## Figures and Tables

**Table 1:** Vignette design.

Design Group	Vignettes	Condition Sets	Checklist Items per Vignette (Mean)	Conflict-Resolution Items (Mean)
Single-condition	24	6	6.4	0.0
Concordant pair	8	2	9.2	0.0
Conflicting pair	32	8	12.2	1.1
Triple-condition	12	3	16.7	2.0

**Table 2:** Worked example: a shortened conflicting-pair vignette, its computed targets and checklist, and the graded response of one model.

Element	Content
Vignette (shortened)	Woman, 46 years, 156 cm, 91.5 kg, BMI 37.6; obesity and CKD (eGFR 35 mL/min/1.73 m^2^, potassium 4.2 mEq/L, phosphorus 4.8 mg/dL, albumin 3.4 g/dL); losartan 50 mg daily; lightly active
Computed targets	Energy 1668–1868 kcal/day (CKD floor of 25 kcal/kg of adjusted weight 66.7 kg, plus 200 kcal); protein 0.55–0.8 g/kg (37–53 g/day); sodium < 2000 mg/day; limit high-potassium foods and monitor potassium (losartan)
Must-do, critical	Protein 0.55–0.8 g/kg (37–53 g/day)
Must-do, critical	Energy 1668–1868 kcal/day, with attention to preventing malnutrition
Must-do, critical	Warns about high-potassium foods and supplements with the angiotensin receptor blocker and asks for potassium monitoring
Must-not, critical	No high-protein diet, protein powder, or potassium or herbal supplements
Conflict resolution, critical	CKD protein limit instead of the higher obesity target, with gradual weight loss and monitoring for malnutrition
Conflict resolution, critical	Energy within 1668–1868 kcal/day without rapid weight loss
Response excerpt 1 (GPT-6 Luna, English)	Protein target: “About 50 g/day (approximately 0.8 g/kg of reference/ideal weight…)”; foods to avoid: “Avoid high-protein diets, protein powders…”
Panel verdicts, excerpt 1	Protein item and protein conflict item: yes (3 of 3 judges)
Response excerpt 2 (same response)	Energy target: “About 1500 kcal/day to support gradual weight loss…”
Panel verdicts, excerpt 2	Energy item and energy conflict item: no (votes no, partial, no); safety-critical flag by 2 of 3 judges for an energy target below the computed range
Response scores	Weighted coverage 0.68; conflict-resolution score 0.50 (one of two items); safety-critical flag: yes

**Table 3:** Evaluated models.

Model	Group	Developer	Access	Model Identifier	Reasoning Setting
GPT-6.1 Sol	Proprietary, flagship	OpenAI	OpenAI API	gpt-6.1-sol	low
Claude Opus 5.5	Proprietary, flagship	Anthropic	Anthropic API	claude-opus-5-5	low
Gemini 3.1 Pro	Proprietary, flagship	Google	Google API	gemini-3.1-pro-preview	low
GPT-6 Luna	Proprietary, economy	OpenAI	OpenAI API	gpt-6-luna	low
Gemini 3.8 Flash	Proprietary, economy	Google	Google API	gemini-3.8-flash	low
Llama 3.3 70B	Open-weight	Meta	Together AI API	meta-llama/Llama-3.3-70B-Instruct-Turbo	n/a
DeepSeek V4 Pro	Open-weight	DeepSeek	Together AI API	deepseek-ai/DeepSeek-V4-Pro-0813	default
Trendyol-LLM-7B	Turkish-adapted open-weight	Trendyol	Local (Hugging Face)	Trendyol/Trendyol-LLM-7B-chat-v4.1.0	n/a
Cosmos-8B	Turkish-adapted open-weight	YTU COSMOS	Local (Hugging Face)	ytu-ce-cosmos/Turkish-Llama-8b-Instruct-v0.1	n/a

**Table 4:** Checklist coverage by model and language.

Model	Weighted Coverage EN	Weighted Coverage TR	Critical Coverage EN	Critical Coverage TR	Conflict Resolution EN	Conflict Resolution TR
GPT-6.1 Sol	0.90 (0.88–0.92)	0.92 (0.90–0.94)	0.91	0.93	0.89	0.86
Claude Opus 5.5	0.91 (0.89–0.93)	0.90 (0.88–0.92)	0.91	0.90	0.80	0.83
Gemini 3.1 Pro	0.81 (0.79–0.83)	0.83 (0.80–0.86)	0.86	0.86	0.67	0.64
GPT-6 Luna	0.87 (0.85–0.89)	0.87 (0.85–0.89)	0.92	0.91	0.75	0.66
Gemini 3.8 Flash	0.85 (0.83–0.87)	0.85 (0.83–0.87)	0.87	0.88	0.64	0.57
Llama 3.3 70B	0.50 (0.48–0.53)	0.44 (0.42–0.46)	0.55	0.49	0.05	0.00
DeepSeek V4 Pro	0.86 (0.84–0.88)	0.86 (0.84–0.88)	0.89	0.88	0.64	0.73
Trendyol-LLM-7B	0.27 (0.25–0.30)	0.26 (0.24–0.28)	0.31	0.31	0.00	0.00
Cosmos-8B	0.39 (0.36–0.42)	0.26 (0.24–0.29)	0.43	0.29	0.00	0.00

**Table 5:** Language and design-group effects on weighted coverage.

Term	Estimate	95% CI Low	95% CI High	*p*	MixedLM Estimate (Vote Level)	MixedLM 95% CI
Turkish (vs. English)	−0.004	−0.021	0.013	0.641	−0.017	−0.028 to −0.007
Concordant pair (vs. single)	−0.056	−0.099	−0.012	0.012	−0.036	−0.202–0.129
Conflicting pair (vs. single)	−0.001	−0.037	0.036	0.973	−0.011	−0.121–0.099
Triple-condition (vs. single)	0.004	−0.028	0.037	0.791	−0.010	−0.152–0.132
Turkish × concordant pair	−0.005	−0.029	0.019	0.677	0.006	−0.012–0.025
Turkish × conflicting pair	−0.028	−0.046	−0.009	0.004	−0.024	−0.036 to −0.011
Turkish × triple-condition	−0.027	−0.048	−0.005	0.015	−0.017	−0.032 to −0.003

**Table 6:** Language gap per model (English minus Turkish, paired by vignette).

Model	n	Gap, Weighted Coverage (EN − TR)	*p* (Wilcoxon)	*p* (Holm)	Gap, Conflict Resolution (EN − TR)
GPT-6.1 Sol	76	−0.021 (−0.037 to −0.005)	0.013	0.093	0.023
Claude Opus 5.5	76	0.007 (−0.008–0.021)	0.403	1.000	−0.034
Gemini 3.1 Pro	76	−0.018 (−0.039–0.005)	0.108	0.651	0.034
GPT-6 Luna	76	0.000 (−0.021–0.021)	0.954	1.000	0.091
Gemini 3.8 Flash	76	0.004 (−0.015–0.024)	0.438	1.000	0.068
Llama 3.3 70B	76	0.063 (0.035–0.092)	<0.001	<0.001	0.045
DeepSeek V4 Pro	76	0.008 (−0.010–0.028)	0.654	1.000	−0.091
Trendyol-LLM-7B	76	0.011 (−0.016–0.038)	0.443	1.000	0.000
Cosmos-8B	76	0.128 (0.100–0.157)	<0.001	<0.001	0.000

**Table 7:** Safety-critical flags by category, model and language (share of responses, %).

Model	Clinical Hazard EN	Clinical Hazard TR	Target Deviation EN	Target Deviation TR
GPT-6.1 Sol	0.0	3.9	5.3	2.6
Claude Opus 5.5	1.3	2.6	2.6	3.9
Gemini 3.1 Pro	11.8	13.2	5.3	6.6
GPT-6 Luna	2.6	3.9	2.6	1.3
Gemini 3.8 Flash	11.8	22.4	2.6	5.3
Llama 3.3 70B	30.3	52.6	31.6	25.0
DeepSeek V4 Pro	14.5	19.7	6.6	10.5
Trendyol-LLM-7B	60.5	65.8	11.8	30.3
Cosmos-8B	59.2	72.4	31.6	31.6

**Table 8:** Cultural appropriateness of Turkish responses (1–5).

Model	Availability	Portion Units	Cuisine Fit	Substitution	Ramadan Adaptation	Mean of 4 Dimensions (95% CI)
GPT-6.1 Sol	4.99	5.00	4.99	4.99	4.97	4.99 (4.98–5.00)
Claude Opus 5.5	4.96	5.00	4.99	4.94	4.82	4.97 (4.96–4.98)
Gemini 3.1 Pro	4.92	5.00	4.91	4.82	4.74	4.91 (4.88–4.94)
GPT-6 Luna	4.94	4.98	4.93	4.94	4.72	4.95 (4.92–4.97)
Gemini 3.8 Flash	4.91	5.00	4.96	4.81	4.86	4.92 (4.90–4.94)
Llama 3.3 70B	3.78	4.31	3.14	2.78	2.52	3.50 (3.39–3.61)
DeepSeek V4 Pro	4.82	5.00	4.84	4.73	4.89	4.85 (4.81–4.88)
Trendyol-LLM-7B	3.16	1.80	2.52	1.71	1.15	2.30 (2.20–2.41)
Cosmos-8B	3.40	3.71	2.73	1.89	1.35	2.94 (2.82–3.05)

## Data Availability

The data presented in this study are available on request from the corresponding author.

## Supplementary Materials

The following supporting information can be downloaded at: https://www.mdpi.com/article/10.3390/nu18213674/s1, Table S1: Output limits, response lengths and truncated responses by model and language; Table S2: Language and design-group effects on weighted coverage in the 54 vignettes with identical case text; Table S3: Language gap per model on the 54 vignettes with identical case text (English minus Turkish); Table S4: Language and design-group interactions in five sensitivity analyses.

## Abbreviations

The following abbreviations are used in this manuscript:

CI	confidence interval
CKD	chronic kidney disease
HTN	hypertension
ICC	intraclass correlation coefficient
LLM	large language model
MNT	medical nutrition therapy
T2DM	type 2 diabetes mellitus
