# Supplementary material for: Large Language Model Dietary Recommendations for Multimorbidity with Conflicting Guidelines in Turkish and English
Source: Nutrients. 2026 Nov 5;18(21):3674. doi: 10.3390/nu18213674 (PMC13765866; doi:10.3390/nu18213674)
Supplement: Supplementary file 1 [file nutrients-18-03674-s001.zip › nutrients-4651553-supplementary.pdf]

Supplementary Materials

Large Language Model Dietary Recommendations for Multimorbidity with Conflicting Guidelines in Turkish and English

Büşra Göz, Yılmaz Dikilitaş, Furkan Göz and Ahmet Sayar

Table S1. Output limits and truncated responses

Table S1 lists, for every model and language, the output limit, the median and longest output and the number of responses that reached the limit. Token counts come from the usage records of the application programming interfaces, and the Google interface does not report output tokens, so the Gemini rows give character lengths only. A response counted as truncated when the interface reported that generation stopped at the limit. Ten of the 1368 responses were truncated, and no proprietary model reached its limit.

Table S1: Output limits, response lengths and truncated responses by model and language

**Model**	**Language**	**Output limit (tokens)**	**Median output (tokens)**	**Max output (tokens)**	**Median length (characters)**	**Truncated responses**
GPT-6.1 Sol	EN	5000	1928	2521	7319	0
GPT-6.1 Sol	TR	5000	2359	3106	6655	0
Claude Opus 5.5	EN	9000	4797	6929	7970	0
Claude Opus 5.5	TR	9000	6078	8751	7887	0
Gemini 3.1 Pro	EN	5000	n/a	n/a	4604	0
Gemini 3.1 Pro	TR	5000	n/a	n/a	4617	0
GPT-6 Luna	EN	5000	1850	2302	5761	0
GPT-6 Luna	TR	5000	2396	3159	5657	0
Gemini 3.8 Flash	EN	5000	n/a	n/a	6394	0
Gemini 3.8 Flash	TR	5000	n/a	n/a	5541	0
Llama 3.3 70B	EN	5000	1053	1316	4434	0
Llama 3.3 70B	TR	5000	1220	1520	3567	0
DeepSeek V4 Pro	EN	11000	7309	14659	5453	1
DeepSeek V4 Pro	TR	11000	6109	10902	6849	0
Trendyol-LLM-7B	EN	3000	593	3000	1735	6
Trendyol-LLM-7B	TR	3000	735	3000	1855	1
Cosmos-8B	EN	3000	785	1080	2953	0
Cosmos-8B	TR	3000	691	3000	1897	2

Table S2. Regression on the 54 vignettes with identical case text

Table S2 repeats the primary regression of Section 2.5 on the 972 responses to the 54 vignettes whose Turkish and English case texts were identical, that is, without the 22 gout vignettes whose flare histories differed. Standard errors are clustered by vignette. The average Turkish–English difference from the same model without interaction terms was −0.016 (−0.026 to −0.006, $p = 0.001$).

Table S2: Language and design-group effects on weighted coverage in the 54 vignettes with identical case text

**Term**	**Estimate**	**95% CI low**	**95% CI high**	**p**
Turkish (vs English)	−0.002	−0.021	0.018	0.861
Concordant pair (vs single)	−0.045	−0.090	−0.001	0.045
Conflicting pair (vs single)	−0.002	−0.048	0.044	0.927
Triple-condition (vs single)	0.023	−0.014	0.060	0.223
Turkish × concordant pair	−0.007	−0.034	0.019	0.581
Turkish × conflicting pair	−0.028	−0.050	−0.005	0.017
Turkish × triple-condition	−0.027	−0.054	−0.001	0.043

Table S3. Language gap per model on the 54 vignettes

Table S3 gives the paired language gap in weighted coverage for each model on the same 54 vignettes, tested with the Wilcoxon signed-rank test and adjusted with the Holm procedure over the nine models. Positive values favor English.

Table S3: Language gap per model on the 54 vignettes with identical case text (English minus Turkish)

Model	n	Gap, weighted coverage (EN − TR)	p (Wilcoxon)	p (Holm)
GPT-6.1 Sol	54	−0.021	0.057	0.400
Claude Opus 5.5	54	0.008	0.317	1.000
Gemini 3.1 Pro	54	−0.024	0.068	0.406
GPT-6 Luna	54	−0.011	0.291	1.000
Gemini 3.8 Flash	54	0.005	0.565	1.000
Llama 3.3 70B	54	0.042	0.017	0.134
DeepSeek V4 Pro	54	0.003	0.939	1.000
Trendyol-LLM-7B	54	0.010	0.607	1.000
Cosmos-8B	54	0.132	<0.001	<0.001

Table S4. Sensitivity of the language and design-group interactions

Table S4 collects the two interaction terms of the primary regression, Turkish × conflicting pair and Turkish × triple-condition, from the main analysis and five sensitivity analyses described in Section 2.5. All models include language, design group, their interaction and model, with standard errors clustered by vignette.

Table S4: Language and design-group interactions in five sensitivity analyses

Analysis	Turkish × conflicting pair (95% CI)	p	Turkish × triple-condition (95% CI)	p
Main analysis, all 1368 responses	−0.028 (−0.046 to −0.009)	0.004	−0.027 (−0.048 to −0.005)	0.015
Identical case text, 54 vignettes	−0.028 (−0.050 to −0.005)	0.017	−0.027 (−0.054 to −0.001)	0.043
Without the 10 truncated responses	−0.027 (−0.046 to −0.008)	0.005	−0.026 (−0.048 to −0.005)	0.018
Without the 11 CKD vignettes with an inconsistent creatinine–eGFR pair	−0.030 (−0.051 to −0.009)	0.004	−0.020 (−0.044 to 0.003)	0.081
Unweighted coverage as outcome	−0.030 (−0.050 to −0.010)	0.003	−0.027 (−0.049 to −0.004)	0.023
Critical coverage as outcome	−0.023 (−0.046 to −0.001)	0.040	−0.026 (−0.051 to −0.002)	0.034
